# Hypodermic Use of Stimulants

**Published:** 1883-04-20

**Authors:** T. Curtis Smith

**Affiliations:** Aurora, Indiana


### HYPODERMIC USE OF STIMULANTS.

By T. Curtis Smith, M. D., Aurora, Indiana.

The value of this method of medication in general, stands unquestioned by the great mass of intelligent practitioners.

Twenty years ago, it was unheard of, except by a favored few; now its use is almost universal in the regular ranks of the profession. To deprive us of the hypodermic use of remedies, would be to take away one of our most powerful and effective plans of meeting and combating many forms of diseases or intercurrent complications.

But we do not propose to speak of the general applicability of this plan. Almost every one knows that morphia is discharged from the hypodermic syringe into the tissues of the human body, with, perhaps, greater frequency than all other therapeutic agents combined. To tell the strory of such use of the agent would be but thrashing old straws.

Of the general use of stimulants, hypodermically, less has been written, and possibly less known.

Very often, from the collapse of shock or injury, the sudden onset of some violent disease, the collapse of a heart-stroke, or that which announces the threatened final dissolution from some low form of disease, we feel the need of a rousing stimulant. The demand is imperative. The case is often clearly such that if we can tide it over the present critical state, we may feel assured that it may recover.

But the stomach may be irritable and reject all that is put into it. The nervous depression may be so great that the stomach refuses to absorb quickly—because the nerve centers are paralyzed—stunned. Any stimulant thrown into it is not soon taken up. Its slowness of absorption—almost total inaction—in such states of the system, is well known. Often, to wait on its tardy motion, is to only wait for the pale messenger. By the rectum, the method is no better. It may or may not be retentive. So also the stomach may be full of food, or the food may be fermenting instead of digesting—soured—and utterly refuse to perform the functions as promptly as needed to save life.

What shall we do? Stand still and see precious time go by, and death come without a risisting effort? By no means! In many cases, it were useless to do otherwise than simply seem to do something until death has completed its work, because we cannot do more than this. But very often the case stands otherwise, and here the value of stimulants comes to our aid by the hypodermic method. Morphia may often be our best stimulant, especially if the depression is largely the result of pain. Combined with atropia, it is often a most powerful stimulant to the circulation and respiration. For instance: I well remember the case of a youth who seemed to be suffering total collapse from an injury received by reason of an explosion of a steam boiler. His friends said:—"Let him alone; he can but die, and we would not add to his sufferings!" I injected a stimulant dose of morphia—he rallied a little—I repeated the dose. The collapse was soon overcome, and he finally made a good recovery. This patient could not swallow, could scarcely breathe, was pallid and cold, yet under morphia, in stimulant doses, twice repeated, rallied and finally recovered.

I have seen quite a number of similar, though less severe cases, rally in a similar manner under the use of morphia, and I think better under morphia and atropia combined.

Recently I was called to a case presenting all the symptoms of sporadic cholera. I do not mean ordinary cholera-morbus. There were the rice-water vomiting and stools, the cramping depression, cold extremities, livid hands, pinched face, etc. To give any remedy by the stomach only increased the vomiting. The case seemed to tend to a rapid fatality. A resort was promptly made to the hypodermic use of morphia and atropia, followed quickly by whisky, and finally of sulphuric ether. Of the whisky, many syringefuls were used, and in the course of two hours several syringefuls of sulph. ether. Ammon. carb. in solution, was given by way of the stomach, sinapisms and heat were used externally. The hypodermic use of the stimulant was all that seemed to produce any noticeable effect; the response to sulphuric ether being far more marked than from any other agent.

Possibly this case might have recovered by other means than the hypodermic syringe; but I do not believe it. I think, now, that it was a mistake to use the alkaline carb. ammon. internally, and it would have been better practice to give arom. sulph. acid freely, well diluted, for reasons every one must know on second thought.

Again, I was called a few years ago to a case of cholera; found it in the algid stage, with death near at hand. Some prompt measure of stimulation must at once avail for him, or he would succumb speedily and surely. Everything swallowed had been rejected; not even a teaspoonful of water could be taken without provking emesis. A hypodermic injection of 1.30 grain of atropia was given. In a half hour, the skin began to warm up, and became florid. He could retain water, and in a short time retained fluid food. He rallied promptly and recovered. A very competent physician had used his endeavor to rally him, and so far had utterly failed. The hypodermic stimulant had roused his nerve centers, especially those of respiration and circulation, and this secured better general vital action and a start toward recovery.

I have seen cases of very severe cholera morbus that would have had a sure but slow re-action from prostration, brought quickly up by the hypodermic use of atropia.

But in the use of this agent, one must be careful not to overdo the matter. No case demands the 1–30 grain at once, unless the prostration is extreme, and a very powerful appeal to the controlling nerve centers must be had immediately in order to save life. This dose should not be repeated in any case, unless it be one of opium-poisoning, and then very cautiously. A too long-continued or too powerful stimulation with atropia, brings a secondary depression of the nerve centers that will quite certainly prove fatal.

We have a most powerful remedy in whisky for hypodermic use. This may be given alone, or combined with carbonate of ammonia. The whisky alone rarely causes any ulceration; but combined with ammonia is very liable to do so, if there is more than about a grain to a drachm. A half grain to the drachm is enough, and then, if needed, several syringefuls can be given at different points.

A weak, aqueous solution of carbonate of ammonia is also a very powerful stimulant, and will be available to counteract conditions of depressions from shock or disease.

In December, 1881, I attended a case of typhoid fever where the depression became so great that the youth was thought to be dying. He was insensible, and the pulse barely perceptible, respiration irregular and shallow, feet cold, etc. I thought the case quite hopeless. He could not swallow, and a little water put into his mouth threatened suffocation. Injected several syringefuls of whisky, with as much carbonate of ammonia as it would hold in solution. In a little time, the pulse began to fill up, the skin assumed a better color, feet warmed, and he became able to swallow. From that time, he was treated to the free use of stimulants by the stomach, though he had had these before, and made a good though slow recovery. There were two ulcers at points where the hypodermics were used, but I thought these were better than the funeral which seemed to stare us in the face at one time.

I wish to refer more especially now to the hypodermic use of sulphuric ether as a stimulant. I have never known it to produce any ulceration or evil effects whatever, though it may produce abscess, or be given in such quantities as to overwhelm the brain, and result badly, just as an overdose by the stomach would readily and quickly do. I know of no agent we can place under the skin that will so promptly and surely bring a cardiac and respiratory response as sulph. ether in about a thirty minim dose. This can be repeated in a half hour if needed, and in very urgent cases the dose may be larger. The immediate local sensation of a hypodermic injection of this agent is often like that of a quick stroke of the actual cautery. This passes quickly away. In many cases, no such sensation is felt. The next noticeable effect will be that as Dupuy says: "The pulse rapidly gains, both in fullness and strength; the heart beats—scarcely perceptible—become more distinct; often, after one or two injections, cyanosis and collapse disappear, and the patient awakens from a condition which was considered desperate."

After very great hemorrhage, Dupuy, of France, Hecker, of Germany, and Parvin, of Indianapolis, speak highly of its use in the low prostration resulting from puerpural hemorrhage. The first named commends it highly in collapse ; from almost any form of hemorrhage or shock from injury or disease.

My own limited use of it by this method teaches me that in sulphuric ether, hypodermically used, we have a most powerful and fairly safe remedy, in a large proportion of cases, where an immediate and very prompt stimulant is demanded, and where the same is not practicable to give by the mouth. It is less apt to produce abscess than ammonia; more prompt in its effect than whisky alone, or combined with ammonia. Whether it will be equal to, or better, than atropia, in the profound collapse of cholera and like diseases, I cannot say from experience.—*Western Medical Reporter.*

